# MiR-99a alleviates apoptosis and extracellular matrix degradation in experimentally induced spine osteoarthritis by targeting FZD8

**DOI:** 10.1186/s12891-022-05822-8

**Published:** 2022-09-20

**Authors:** Yeyang Wang, Xiaoyu Zheng, Dixin Luo, Wangyang Xu, Xiaozhong Zhou

**Affiliations:** 1 The Spine Department, Orthopaedic Center, Guangdong Second Provincial General Hospital, Guangzhou, Guangdong Province China; 2 The Second Clinical School, Southern Medical University, Guangzhou, China; 3 Guangdong Medical University, Zhanjiang, Guangdong Province China

**Keywords:** Osteoarthritis, miR-99a, FZD8, Apoptosis, Extracellular matrix degradation

## Abstract

**Background:**

Our previous study identified miR-99a as a negative regulator of early chondrogenic differentiation. However, the functional role of miR-99a in the pathogenesis of osteoarthritis (OA) remains unclear.

**Methods:**

We examined the levels of miR-99a and Frizzled 8 (FZD8) expression in tissue specimens. Human SW1353 chondrosarcoma cells were stimulated with IL-6 and TNF-α to construct an in vitro OA environment. A luciferase reporter assay was performed to analyze the relationship between miR-99a and *FZD8*. CCK-8 assays, flow cytometry, and ELISA assays were used to assess cell viability, apoptosis, and inflammatory molecule expression, respectively. Percutaneous intra-spinal injections of papain mixed solution were performed to create an OA Sprague–Dawley rat model. Alcian Blue staining, Safranin O Fast Green staining, and Toluidine Blue O staining were performed to detect the degrees of cartilage injury.

**Results:**

MiR-99a expression was downregulated in the severe spine OA patients when compared with the mild spine OA patients, and was also decreased in the experimentally induced in vitro OA environment when compared with the control environment. Functionally, overexpression of miR-99a significantly suppressed cell apoptosis and extracellular matrix degradation stimulated by IL-6 and TNF-α. *FZD8* was identified as a target gene of miR-99a. Furthermore, the suppressive effects of miR-99a on cell injury induced by IL-6 and TNF-α were reversed by FZD8 overexpression. Moreover, the levels of miR-99a expression were also reduced in the induced OA model rats, and miR-99a agomir injection relieved the cartilage damage. At the molecular level, miR-99a overexpression downregulated the levels of MMP13, β-catenin, Bax, and caspase-3 protein expression and upregulated the levels of COL2A1 and Bcl-2 protein expression in the in vitro OA-like chondrocyte model and also in the experimental OA model rats.

**Conclusions:**

Our data showed that miR-99a alleviated apoptosis and extracellular matrix degradation by targeting FZD8, and thereby suppressed the development and progression of experimentally induced spine osteoarthritis.

**Supplementary Information:**

The online version contains supplementary material available at 10.1186/s12891-022-05822-8.

## Background

Osteoarthritis (OA) is a chronic degenerative joint disease characterized by degradation of articular cartilage, synovitis, joint inflammation, and subchondral bone remodeling [1, 2]. Chondrocytes are the only cells in articular cartilage capable of maintaining cartilage homeostasis and integrity by producing extracellular matrix proteins [3]. It has been reported that pro-inflammatory cytokines and mechanical stress-induced molecular events that cause chondrocyte damage are correlated with OA development [4–7]. Therefore, gaining a better understanding of the molecular mechanism of chondrocyte injury may help us to develop molecular targeted drugs for OA.

MicroRNAs (miRNAs/miRs) comprise a group of endogenous, small non-coding RNAs that are 19–25 nucleotides in length and regulate gene expression by binding to the 3′-untranslated regions (3′-UTRs) of specific target gene mRNA molecules [8]. Several miRNAs involved in the inflammatory response, chondrogenesis, and cartilage remodeling are crucial for OA progression [9, 10]. For example, an upregulation of miR-10a-5p expression in OA is known to trigger apoptosis in chondrocytes by targeting HOXA1 [11]. It was shown that the interleukin-1 beta (IL-1β)-induced inflammatory response and cartilage matrix degradation are both alleviated when miR-613 is overexpressed in CHON-001 cells [12]. Additionally, miR-204-5p was shown to target Runt-related transcription factor-2 (Runx2) to inhibit chondrocyte proliferation and development of an OA-like phenotype in rats [13]. MiR-99a, located on chromosome 21q21.1, was reported to potentially regulate adipose tissue inflammation [14]. MiR-99a was recently reported to act as a tumor suppressor or oncogene by regulating cell proliferation and apoptosis in various cancers [15–17]. A recent RNA sequencing study found that miR-99a was downregulated in the lesions of OA articular cartilage when compared with preserved cartilage; furthermore, miR-99a was verified to correlate with the expression of certain genes (*WNT9A*, *FZD1,* and *GDF6*), that help to regulate OA [18]. Our previous study showed that miR-99a directly targets bone morphogenetic protein receptor type 2 (BMPR2) to suppress early chondrogenic differentiation [19]. Accordingly, it may be speculated that miR-99a acts a key regulator in the initiation and progression of OA.

Frizzled 8 (FZD8), a member of the Frizzled (FZD) receptor family, plays a positive role in regulating canonical and non-canonical Wnt/β-catenin pathways [20]. As a tissue repair and fibrosis-related regulator, the canonical Wnt/β-catenin pathway is highly activated in OA-derived human synovial fibroblasts [21]. Overexpression of β-catenin significantly promotes chondrocyte hypertrophy, cartilage degeneration, and matrix protease activity within mature chondrocytes [22–25]. A bioinformatics analysis revealed that miR-99a has putative binding sites for FZD8 mRNA. However, the biological role of the miR-99a/FZD8 axis in OA remains unclear.

In this study, we investigated the effects of miR-99a in an in vitro OA-like chondrocyte model and also in an experimentally induced rat OA model in vivo*.* Our data further confirmed an association between miR-99a and FZD8 in the in vitro OA-like chondrocyte model. Moreover, we evaluated whether miR-99a played a functional role in the in vitro OA-like chondrocyte model by targeting FZD8.

## Methods

### Patients and specimens

Samples of human spine facet joint tissue were obtained from spinal levels L3-S1 of patients (age range, 42–73 years) diagnosed as spinal osteoarthritis, and who subsequently underwent lumbar surgery because of neurogenic claudication. Patients with L3-S1 lumbar facet joint degradation and no history of spinal surgery were included in this study. Patients with an infection or autoimmune disease were excluded from the study. All spine facet joints were graded according to computed tomography (CT) and T2 magnetic resonance imaging (MRI) results based on the criteria of Weishaupt [26] (Grade 0–3). Next, the Grade 2 facet joints were assigned to a mild OA group and the Grade 3 joints were assigned to a severe OA group (10 samples per group). Grade 2 was defined as narrowing of the facet joint space and/or moderate osteophytes and/or moderate hypertrophy of the articular process and/or mild subarticular bone erosions. Grade 3 was defined as narrowing of the facet joint space and/or large osteophytes and/or severe hypertrophy of the articular process and/or severe subarticular bone erosions and/or subchondral cysts [26].

Corresponding articular cartilage tissues and blood samples collected from the severe and mild spine OA groups were stored at − 80 °C. Two cartilage samples were collected from each patient; one to be used for histology staining and one for quantitative real-time PCR analysis and western blot analysis. The blood samples were used to detect concentrations of inflammatory factors. All enrolled patients provided their written informed consent for study participation.

### Animal studies

A total of 30 male Sprague–Dawley rats (10 weeks old) were provided by the Animal Center of the Chinese Academy of Sciences (Shanghai, China). The rats were housed in polypropylene cages in a room with a 12 h dark/light cycle; food and water were available ad libitum. Next, the rats were randomly assigned to 3 groups: sham, spine OA model, and model + miR-99a, respectively, with 10 rats per group. The OA rat model was induced by giving a percutaneous intra-spinal injection of 20 μL papain mixed solution (0.2 mL of 4% papain mixed with 0.1 mL of 0.03 mol/L l-cysteine) into the second/third lumbar spine each week for 3 consecutive weeks. Briefly, the rats were anaesthetized with isoflurane and their backs were shaved. A 21 G butterfly needle was inserted into a point 5 mm to the right of the middle of the L2 and L3 spinous processes. Next, the butterfly needle was slightly moved to slide into the L2/L3 facet joint. Finally, a microinjector with a 26 G needle was inserted into the butterfly needle, and the papain mixed solution was slowly injected. The left sides of the spinous processes were also treated with papain mixed solution. Rats in the sham group were injected with an equal amount of normal saline solution. After 3 days of injection with papain mixed solution, the rats in the model + miR-99a group received an additional injection of the miR-99a agomir (50 nmol in 100 μL saline) that was administered in the same manner. At 4 weeks post-operation, the rats were euthanized with CO_2_ and serum samples were collected. Additionally, samples of articular cartilage tissue from the second/third lumbar spine were collected and cryopreserved.

### Cartilage tissue staining and analysis

Alcian Blue staining (AB), Safranin O Fast Green staining (SF), and Toluidine Blue O staining (TBO) were performed to detect cartilage injuries. Samples of cartilage tissue were fixed in formaldehyde, decalcified in ethylene diamine-tetra acetic acid (EDTA) solution, embedded in paraffin, and then cut into sections. Next, the sections were stained in Alcian Blue solution for 30 min, or with Toluidine Blue solution for 3 min, respectively; after which, they were washed in water and alcohol and observed under a microscope. The cartilage was stained blue by AB and purple by TBO. As for SF staining, the sections were stained with fast green dyeing solution for 5 min and then with Safranin O for 2 min; after which, the cartilage was stained red. The OARSI score [27] and cartilage thickness were further analyzed based on the SF stained sections. Three sections from each sample were analyzed.

### Enzyme-linked immunosorbent assay (ELISA)

ELISA assays were performed to analyze the pro-inflammatory cytokines in samples of blood serum. In brief, the levels of IL-1β, IL-6 and tumor necrosis factor (TNF)-α expression in serum samples were determined according to instructions provided by manufacturer of each ELISA kit (R&D Systems, Minneapolis, MN, USA).

### Chondrocyte culture and stimulation

SW1353 human bone chondrosarcoma cells were provided by the Institute of Biochemistry and Cell Biochemistry and Cell Biology (Shanghai, China) and subsequently maintained in DMEM (HyClone, Logan UT, USA) supplemented with 10% FBS (Gibco) at 37 °C. Next, a particularly effective cell model of OA cartilage was produced by stimulation with IL-6 and TNF-α. In brief, SW1353 cells were treated with 10 ng/mL IL-6 and TNF-α, and then incubated for 0, 12, 24, 36, 48, and 56 h, respectively.

### Cell transfection

An miR-99a agomir, antagomir, and scramble construct were provided by RiboBio Co., Ltd. (Guangzhou, China). An FZD8 plasmid and empty vector were provided by GENE Company (Shanghai, China). SW1353 cells were seeded into the wells of a 6-well plate (2 × 10^5^ cells per well) and incubated for 24 h at 37 °C in an atmosphere of 95% air. Lipofectamine 2000 (Invitrogen, Carlsbad, CA, USA) was used to transfect the cells with 50 nM oligonucleotides and/or 100 nM plasmids for 48 h; after which, the cells were stimulated with IL-6 and TNF-α for 48 h.

### CCK-8 assay

To analyze cell viability, Cell Counting Kit-8 assays were conducted according to the manufacturer’s protocol (CCK-8; Dojindo Molecular Laboratories, Inc.). Briefly, 5 × 10^3^ cells were seeded into each well of 96-well plates and cultured at 37 °C in a 95% air atmosphere. Next, 10 µL of CCK-8 reagent dispersed in fresh complete media was added to the cells at 0, 12, 24, 36, 48, and 56 h, respectively; after which, the cells were incubated for an additional 2 h at 37 °C. Finally, a microplate reader was used to determine the absorbance of each well at 450 nm.

### Flow cytometry assay

Cell apoptosis was measured by using flow cytometry and an Annexin V-FITC apoptosis detection kit (Keygen, China) Briefly, cells were harvested using 0.25% trypsin without EDTA and then resuspended in 500 µL of binding buffer. The cells were then incubated with 2 µL of 20 μg/mL Annexin V-FITC and 2 µL of 50 μg/mL propidium iodide (PI) for 15–20 min in the dark, and the apoptotic cells were detected by flow cytometry (BD FACSCalibur, San Jose, CA, USA).

### Immunofluorescence staining

For analysis of FZD8 in cells, SW-1353 cells were seeded onto coverslips in 6-well plates (1 × 10^5^ cells per well) and cultured for 48 h. After 30 min of fixation in 4% paraformaldehyde, the cells were permeabilized with 0.5% TritonX-100 for 10 min. Next, the cells were blocked with 1% BSA in PBS for 2 h and subsequently incubated overnight at 4 °C with anti-FZD8 (1:100, SAB4503133, Sigma-Aldrich St. Louis, MO, USA), followed by incubation with a fluorescent Goat polyclonal Secondary Antibody to Rabbit IgG (1:200, ab150079). The cells were then washed twice with PBS, and DAPI was used to stain the nuclei. Images of the stained cells were acquired using a fluorescence microscope. Red fluorescence indicated FZD8 expression and the nuclei stained blue.

### Quantitative real-time PCR analysis

Total RNA was extracted from tissue samples and cells by using TRIzol reagent (Invitrogen, Carlsbad, CA, USA) according to the manufacturer’s instructions. Next, one microgram sample of total RNA was reverse transcribed into complementary DNA (cDNA) using a TaqMan miRNA reverse transcription kit (Applied Biosystems, Waltham, MA, USA) and a cDNA synthesis kit (Bio-Rad Laboratories, Inc., Hercules, CA, USA). PCR amplifications were performed on a 7500 real-time PCR system (Applied Biosystems) under the following conditions: 10 min at 95 °C, followed by 40 cycles of 20 s at 95 °C, 30 s at 55 °C, and 30 s at 72 °C. The primer sequences used for amplification are shown in Table 1. The relative expression of each target gene was calculated using the 2-ΔΔCt method [28].

**Table 1:** The primers used for qRT-PCR analysis

Genes	Forward primer (5′-3')	Reverse primer (5’-3’)
GAPDH	TGTTCGTCATGGGTGTGAAC	ATGGCATGGACTGTGGTCAT
FZD8	AGTGGGGTTACCTGTTGGAA	ACTGATTGGGCATGTAGGTG
U6	CTCGCTTCGGCAGCACA	AACGCTTCACGAATTTGCGT
hsa-miR-99a	ACACTCCAGCTGGGAACCCGTAGATCCGATCT	CTCAACTGGTGTCGTGGAGTCGGCAATTCAGTTGAGCACAAGAT

### Western blot analysis

The total proteins in each sample were extracted with RIPA lysis buffer (Beyotime Institute of Biotechnology), and the amount of protein in each extract was quantified using a BCA kit (Beyotime, China). Next, an equal amount of protein from each extract (30 µg) was separated by 10% SDS-PAGE, and the protein bands were transferred onto PVDF membranes (Bio-Rad, USA), which were subsequently blocked with 5% non-fat milk for 2 h at room temperature. The membranes were then incubated with anti-FZD8 (SAB4503133, Sigma-Aldrich,), anti-Bax (ab182734, Abcam, Cambridge, MA, USA), anti-Bcl-2 (ab194583, Abcam), anti-caspase-3 (ab184787, Abcam), anti-Col2A1 (LS-C800256, LifeSpan Biosciences, Seattle, WA, USA), anti-MMP-13 (ab39012, Abcam), anti-β-catenin (ab32572, Abcam), and anti-GAPDH (ab181602, Abcam) antibodies overnight at 4 °C; after which, they were incubated for 2 h at room temperature with an HRP-conjugated secondary antibody. Enhanced chemiluminescence reagent (Forevergen Biosciences, Guangzhou, China) was used to detect the immunostained protein bands, and GAPDH served as a loading control.

### Luciferase reporter assay

The wild-type and mutant 3′-UTR sequences of FZD8 were cloned into a pmirGLO luciferase vector (Promega, Madison, WI, USA) to construct 2 recombinant plasmids: WT 3′-UTR FZD8 and MUT 3′-UTR FZD8, respectively. For the luciferase reporter assay, SW1353 cells were plated into 24-well plates at a density of 6 × 10^4^ cells per well and then co-transfected with the recombinant plasmid (200 ng) and 200 ng of miR-99a mimics or the negative control (NC). Following transfection for 48 h, relative luciferase activity was detected using the Dual-Glo Luciferase assay system (Promega).

### Statistical analysis

All statistical analyses were performed using GraphPad Prism software (version 6.0; GraphPad Software, Inc., San Diego, CA, USA). Statistical results are presented as the mean value ± SD of data obtained from 3 independent experiments conducted with cells or 10 independent experiments conducted using patient or rat samples. Differences between two groups were analyzed using Student’s t-test and differences among multiple groups were analyzed by one-way or two-way ANOVA, followed by Tukey’s post hoc test. A *p*-value < 0.05 was considered to be statistically significant.

## Results

### MiR-99a expression was downregulated in severe spine OA-affected cartilage tissues

**Fig. 1:**
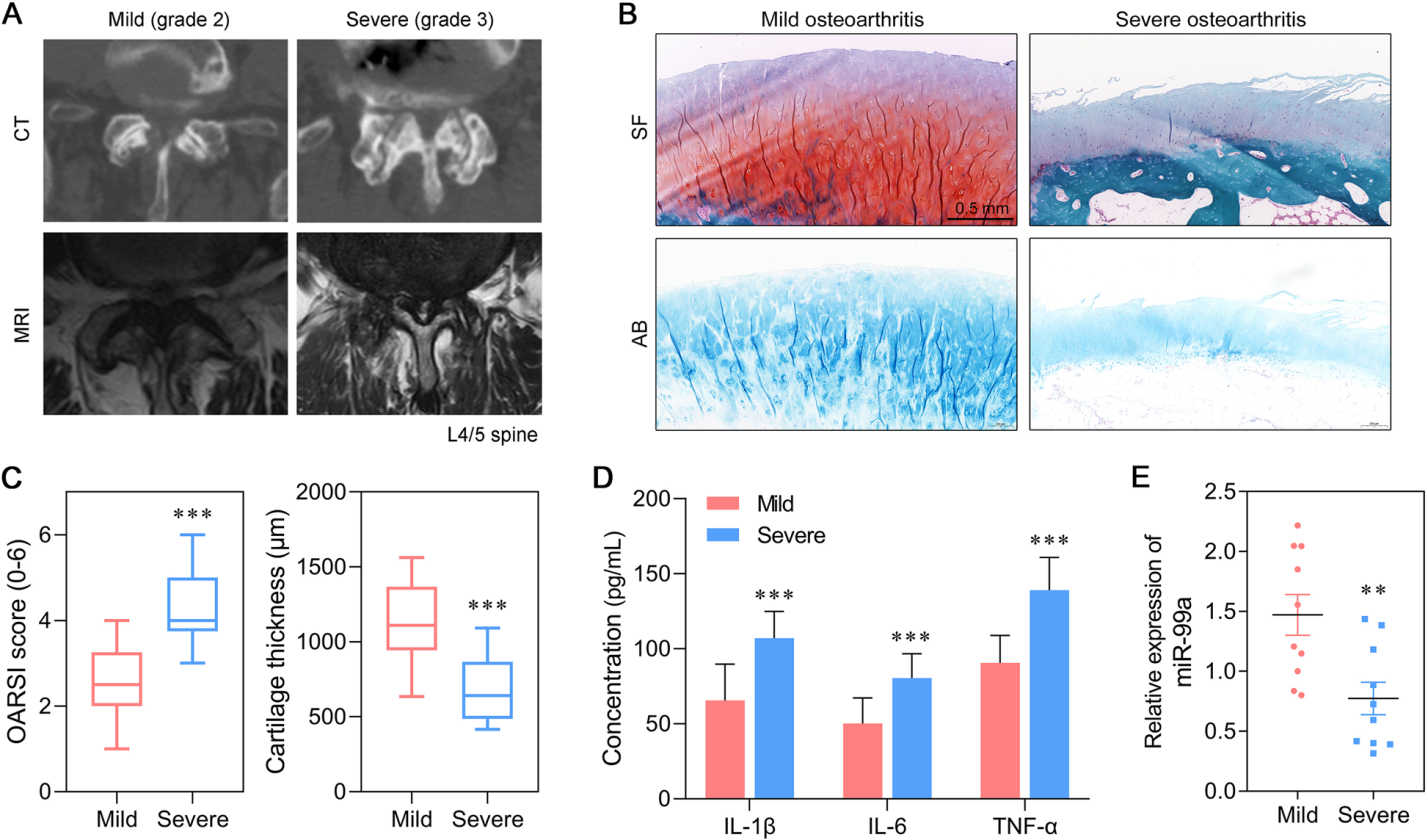
MiR-99a expression was decreased in severe spine OA-affected cartilage tissues. (A) Representative CT and MRI images from the mild group and severe group of patients. (B) Morphological changes in articular cartilage derived from severe spine OA and mild spine OA tissues were evaluated by Safranin O Fast Green staining and Alcian Blue staining. (C) OARSI scores and cartilage thickness were determined by examination of the Safranin O Fast Green staining sections. (D) The levels of IL-1β, IL-6, and TNF-α in severe spine OA tissues and blood samples from mild spine OA patients were analyzed by ELISA. (E) Quantitative real time PCR was used to detect the levels of miR-99a expression in human severe spine OA tissues or mild spine OA joint tissues. **p < 0.01, ***p < 0.001, compared with mild spine OA, *N* = 10. SF, Safranin O Fast Green staining; AB, Alcian Blue staining

Representative CT and MRI images from the mild group and severe group patients are showed in Fig. 1A. We first evaluated the morphological changes that occurred in articular cartilage. As shown in Fig. 1B, Safranin Solid Green staining showed that there were fewer chondrocytes and proteoglycan, and the cartilage layer was thinner in the severe spine OA group than in the mild spine OA group. Moreover, a progressive loss of Alcian Blue staining accompanied by hypocellularity was present in the condylar cartilage layers of the severe spine OA group, when compared with the mild spine OA group (Fig. 1B). The OARSI scores were significantly higher and cartilage thickness was decreased in the severe group when compared with the mild group, indicating differences in the degrees of cartilage injury in the two groups (Fig. 1C). In addition, the levels of inflammatory cytokines were determined by ELISA. Those results showed that the levels of pro-inflammatory cytokines (IL-1β, IL-6, and TNF-α) in blood samples from the severe spine OA group were much higher than those in blood samples from the mild spine OA group (Fig. 1D). Quantitative real time PCR was performed to determine miR-99a expression in the OA-affected cartilage tissues. As shown in Fig. 1E, the levels of miR-99a expression were significantly downregulated in the articular cartilage tissues derived from the severe spine OA group, when compared to articular cartilage tissues derived from the mild spine OA group.

### FZD8 was a potential target of miR-99a

**Fig. 2:**
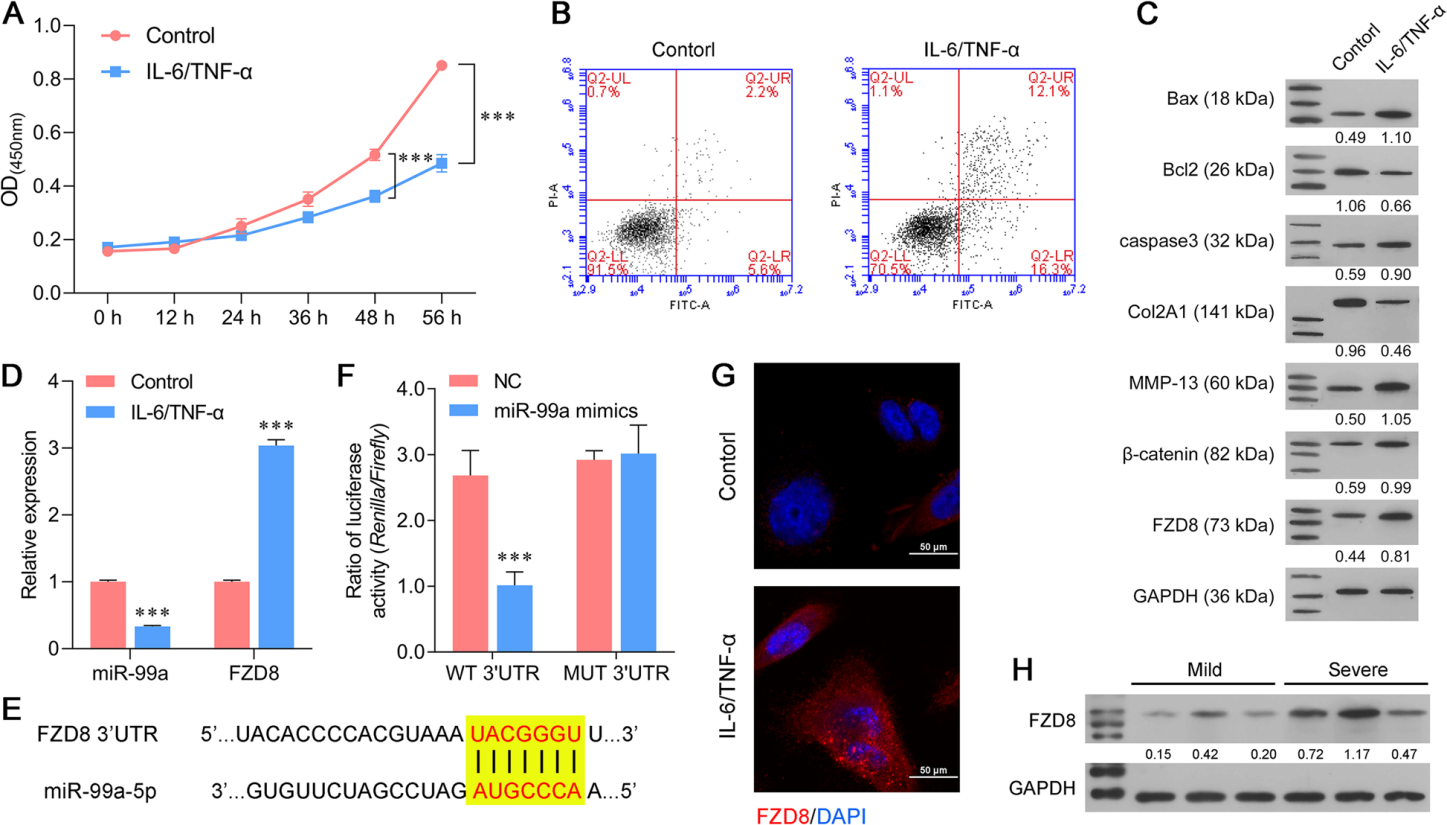
FZD8 was a potential target of miR-99a. (A) The viability of SW1353 cells was determined after 0, 12, 24, 36, 48, and 56 h of treatment with 10 ng/mL IL-6 and TNF-α. (B) SW1353 cell apoptosis was analyzed by flow cytometry. (C) The levels of Bax, Bcl-2, caspase-3, Col2A1, MMP-13, β-catenin, and FZD8 proteins as determined by western blotting. (D) The levels of miR-99a and FZD8 mRNA in SW1353 cells were determined by quantitative real time PCR. (E) The predicted binding sites between miR-99a and the 3′-UTR of FZD8 mRNA. (F) The targeting effect of miR-99a and FZD8 was measured by a luciferase reporter assay. (G) The levels of FZD8 protein in SW1353 cells were detected by immunofluorescence. (H) FZD8 expression in mild OA cartilage tissue and severe OA cartilage tissue as determined by western blotting. ***p < 0.001, compared with control or NC, *N* = 3

Next, SW1353 cells were treated with IL-6 and TNF-α for indicated time periods to induce an in vitro arthritic microenvironment. CCK-8 assays showed a significant reduction in the viability of SW1353 cells treated with IL-6 and TNF-α for 48 consecutive hours (Fig. 2A). Based on CCK-8 assay data, we selected a 48 h of IL-6 and TNF-α treatment in subsequent experiments. Flow cytometry results consistently showed that treatment with IL-6 and TNF-α promoted the apoptosis of SW1353 cells (Fig. 2B). Moreover, we also analyzed the protein markers associated with apoptosis and ECM degradation in SW1353 cells. As shown in Fig. 2C, the levels of Bax, caspase-3, MMP-13 and β-catenin proteins were upregulated, while the levels of Bcl-2 and Col2A1 proteins were downregulated in SW1353 cells treated with IL-6 and TNF-α. Moreover, miR-99a expression was inhibited in the IL-6/TNF-α group (Fig. 2D). A Targetscan database analysis predicted FZD8 as a target of miR-99a, and we therefore investigated the association between miR-99a and FZD8. The predicted binding sites between miR-99a and the 3′-UTR of FZD8 mRNA are depicted in Fig. 2E, and those sites are widely conserved in many species, including the mouse, rat, chimp, and others. Results from luciferase reporter assays showed that when compared to a control reporter, miR-99a suppressed luciferase activity in SW1353 cells that transfected with the WT FZD8 3′-UTR reporter but did not suppress luciferase activity in SW1353 cells that transfected with the MUT FZD8 3′-UTR reporter (Fig. 2F). Furthermore, qPCR and western blot assays showed high levels of FZD8 expression in the IL-6/TNF-α group, which indirectly confirmed the regulatory relationship between miR-99a and FZD8 (Fig. 2C-D). In addition, the levels of FZD8 protein were assessed by immunofluorescence staining; as expected, those levels increased after IL-6/TNF-α simulation (Fig. 2G). We then further detected the expression of FZD8 in samples from the mild and severe spine OA patients, and found that FZD8 expression was higher in severe group (Fig. 2H).

### Overexpression of miR-99a attenuated IL-6/TNF-α‐induced extracellular matrix degradation and cell apoptosis

**Fig. 3:**
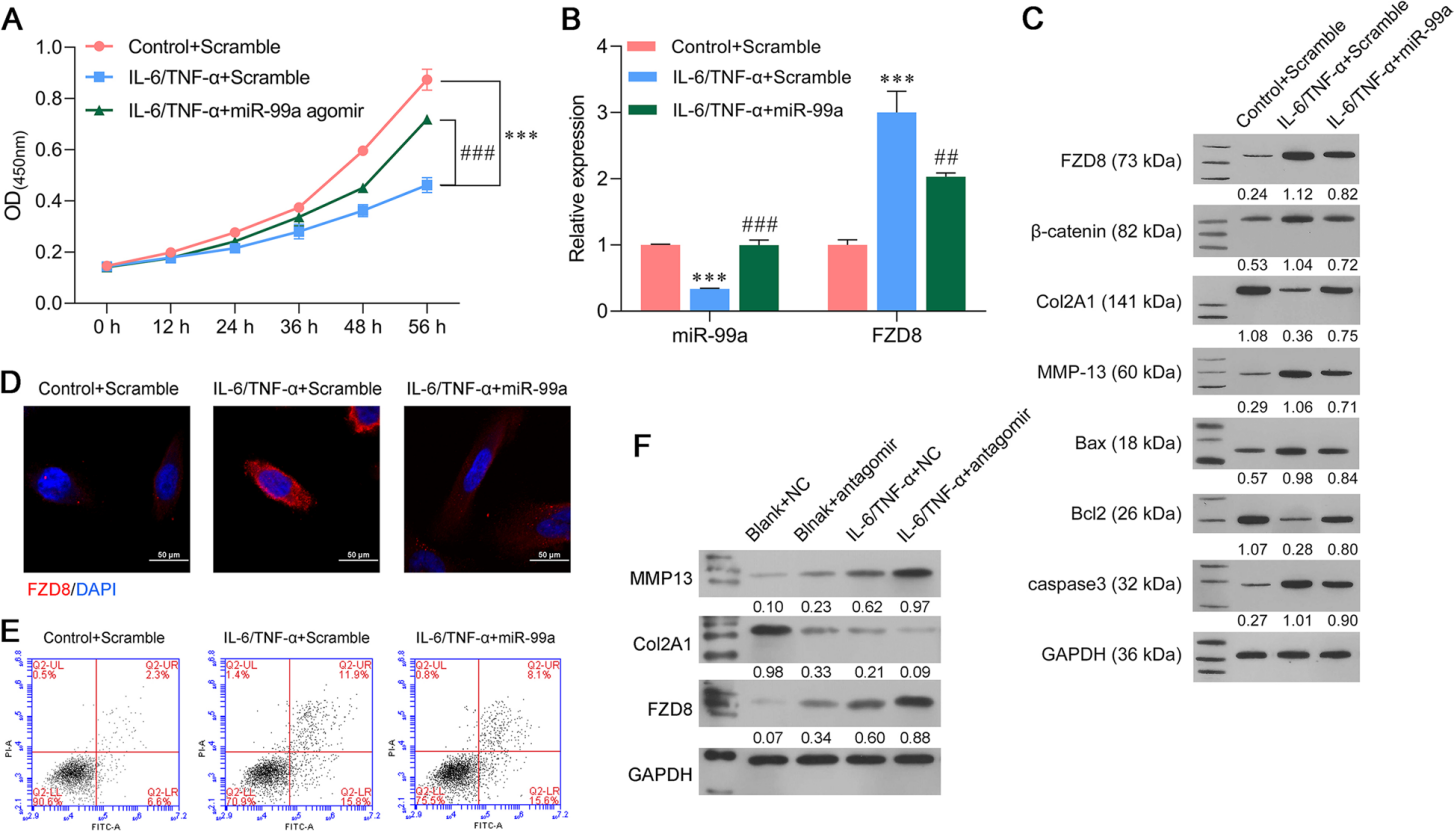
Overexpression of miR-99a attenuated IL-6/TNF-α‐induced ECM degradation and cell apoptosis. SW1353 cells were transfected with the miR-99a agomir, antagomir or scramble and then treated with IL-6 and TNF-α for 48 h. (A) CCK-8 assays were performed to evaluate the viability of transfected SW1353 cells. (B) The levels of miR-99a and FZD8 expression in transfected SW1353 cells. (C) The levels of Bax, Bcl-2, caspase-3, Col2A1, MMP-13, β-catenin, and FZD8 proteins in transfected SW1353 cells were measured by western b lotting. (D) FZD8 protein expression was detected by immunofluorescence. (E) SW1353 cell apoptosis was analyzed by flow cytometry. (F) The levels of MMP13, Col2A1, and FZD8 expression in SW1353 cells transfected with the scramble or antagomir, with or without IL-6/TNF-α stimulation. ***p < 0.001, compared with control + scramble; ##*p* < 0.01, ###*p* < 0.001, compared with IL-6 and TNF-α + scramble, *N* = 3

Because miR-99a expression was downregulated in the in vitro model of OA, we performed gain-of-function assays in SW1353 cells by transfecting the cells with the miR-99a agomir and scramble construct, and then treating them with IL-6 and TNF-α. CCK-8 assay results showed that the decreased viability of IL-6 and TNF-α-stimulated SW1353 cells was significantly reversed after transfection with the miR-99a agomir (Fig. 3A). At the molecular level, quantitative real time PCR analyses demonstrated that transfection with the miR-99a agomir significantly attenuated both the decrease in miR-99a and increase in FZD8 mRNA levels induced by IL-6 and TNF-α stimulation (Fig. 3B). Western blot analyses further confirmed that the IL-6 and TNF-α-induced upregulation of FZD8, β-catenin, MMP-13, Bax, and caspase-3 levels, as well as the downregulation of Col2A1 and Bcl-2 levels were attenuated after miR-99a was overexpressed in SW1353 cells (Fig. 3C). Moreover, the upregulation of FZD8 induced by IL-6/TNF-α stimulation and the downregulation of FZD8 induced by miR-99a were also confirmed by immunofluorescence staining (Fig. 3D). Additionally, the elevated apoptosis rates of IL-6 and TNF-α-stimulated SW1353 cells were attenuated by miR-99a overexpression (Fig. 3E). Finally, to fully explore the function of miR-99a, we also detected the effect of miR-99a inhibition on extracellular matrix degradation. The antagomir was transfected into SW1353 cells, and the cells were then cultured with or without IL-6/TNF-α stimulation. Western blot results showed that regardless of the environment, miR-99a inhibition increased both MMP13 and FZD8 expression and decreased Col2A1 expression (Fig. 3F).

### FZD8 overexpression reversed the effect of miR-99a on IL-6 and TNF-α‐induced extracellular matrix degradation and cell apoptosis

**Fig. 4:**
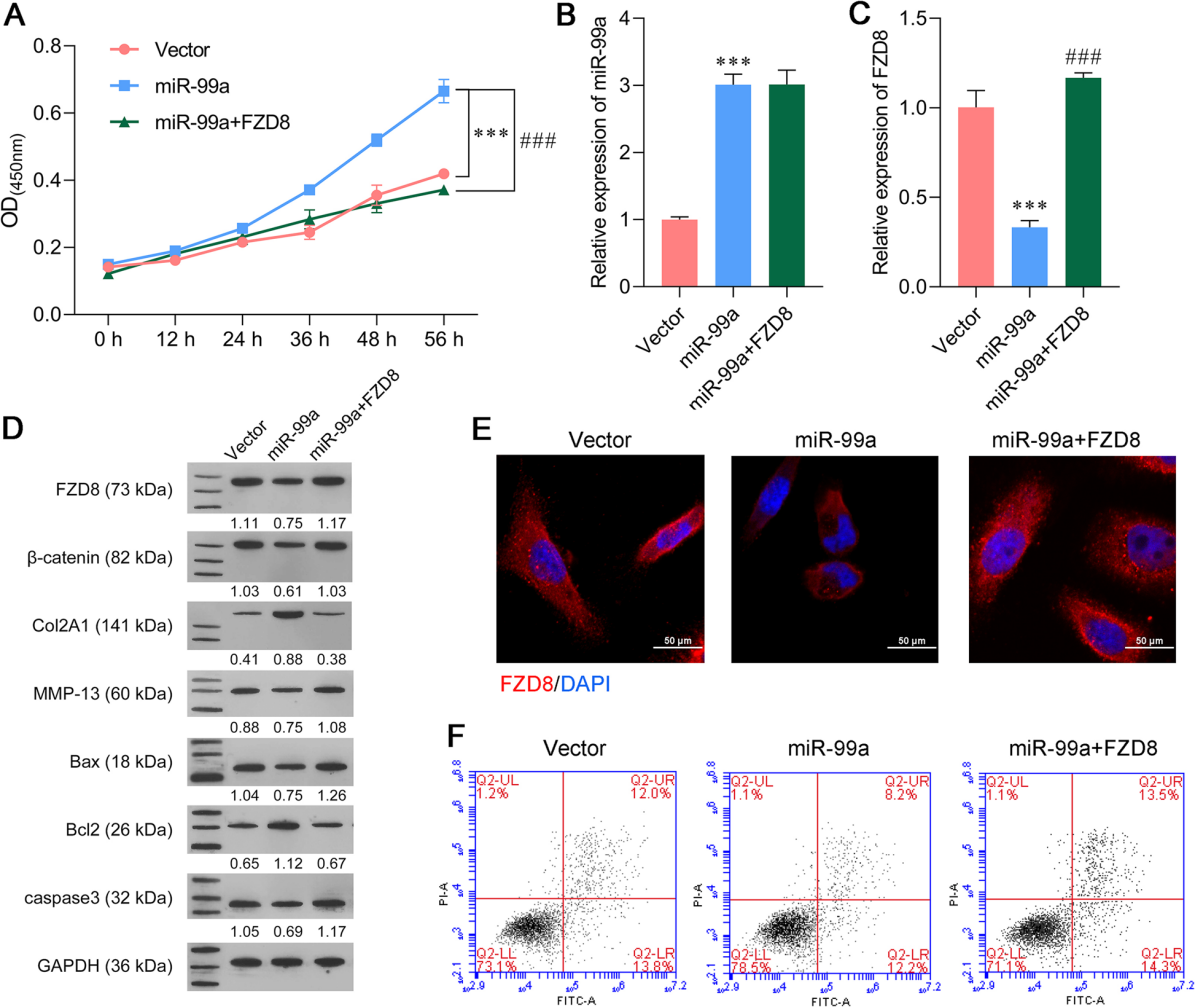
FZD8 overexpression reversed the effects of miR-99a on ECM degradation and apoptosis. SW1353 cells were transfected with the vector, miR-99a agomir alone, or co-transfected with the miR-99a agomir and FZD8 overexpression plasmid, followed by treatment with IL-6 and TNF-α for 48 h. (A) CCK-8 assays were performed to evaluate the viability of transfected SW1353 cells. (B-C) The levels of miR-99a and FZD8 expression were determined in transfected SW1353 cells. (D) The levels of Bax, Bcl-2, caspase-3, Col2A1, MMP-13, β-catenin, and FZD8 proteins were determined by western blotting. (E) The levels of FZD8 protein in transfected SW1353 cells were detected by immunofluorescence. (F) The apoptosis of transfected SW1353 cells was analyzed by flow cytometry. ***p < 0.001, compared with vector; ###*p* < 0.001, compared with miR-99a, *N* = 3

Based on the above results, rescue experiments were performed to determine whether miR-99a exerted its function in IL-6 and TNF-α-stimulated SW1353 cells by targeting FZD8. CCK-8 assays showed that the miR-99a agomir alleviated the IL-6 and TNF-α-induced reduction in cell viability, but that alleviating effect could be subsequently reduced by FZD8 overexpression (Fig. 4A). PCR assays showed that the miR-99a agomir significantly increased miR-99a expression, while overexpression of FZD8 did not affect miR-99a expression (Fig. 4B). Meanwhile, the miR-99a-induced downregulation of FZD8 mRNA levels in IL-6 and TNF-α-stimulated SW1353 cells was notably reversed after co-transfection with the miR-99a agomir and FZD8 overexpression plasmid (Fig. 4C). Western blot assays verified that FZD8 overexpression relieved the effects of miR-99a overexpression on the levels of FZD8, Bax, Bcl-2, caspase-3, Col2A1, MMP-13, and β-catenin proteins in IL-6 and TNF-α-stimulated SW1353 cells (Fig. 4D). Immunofluorescence staining further confirmed the trend of FZD8 protein expression in IL-6 and TNF-α-stimulated SW1353 cells (Fig. 4E). Flow cytometry analyses indicated that the apoptosis of IL-6 and TNF-α-stimulated SW1353 cells was inhibited by the miR-99a agomir, whereas that inhibitory effect was relieved by FZD8 overexpression (Fig. 4F). These findings revealed that miR-99a relieved IL-6 and TNF-α-induced SW1353 cell injuries by targeting FZD8.

### Attenuation of OA progression by miR-99a overexpression

**Fig. 5:**
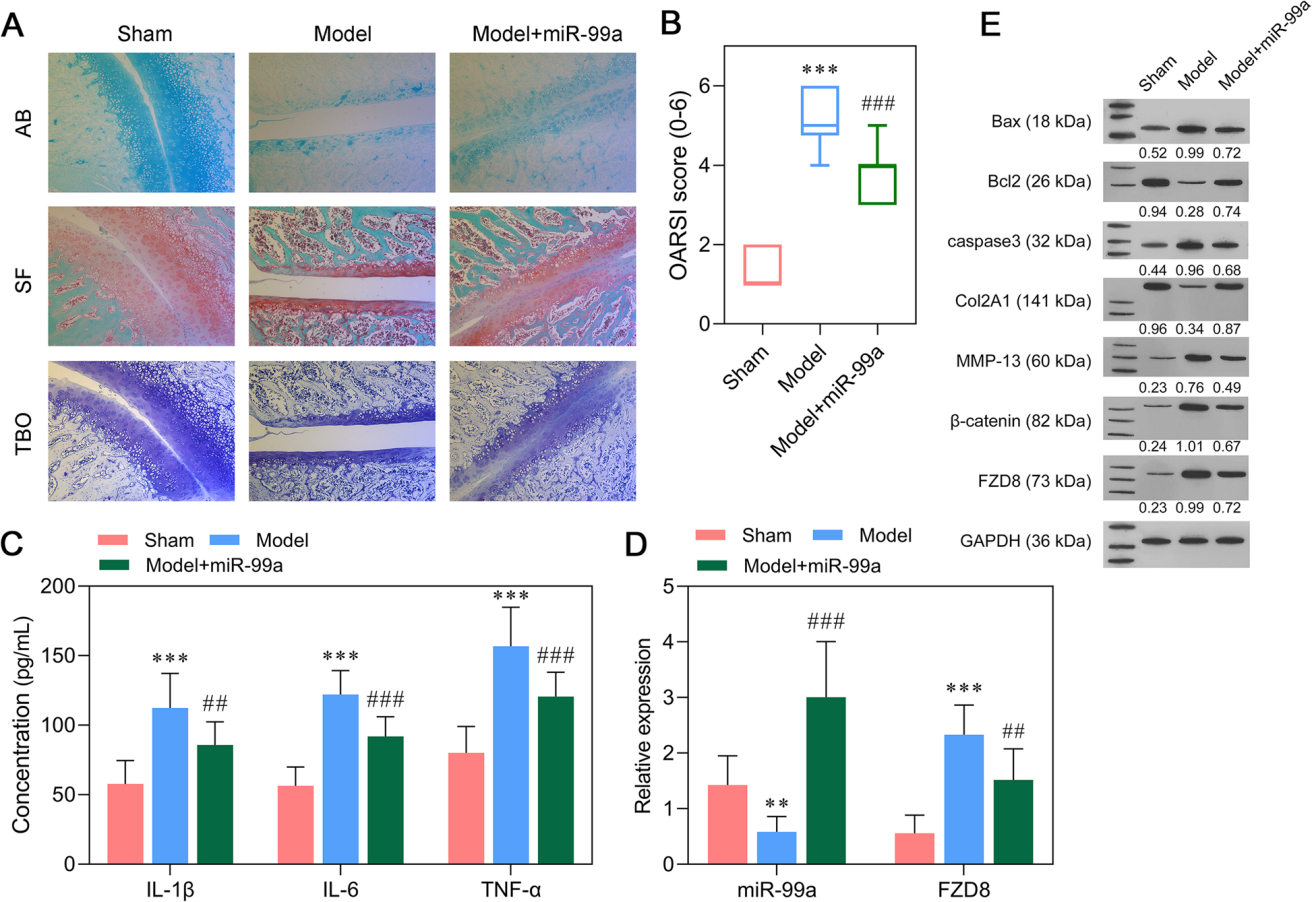
Attenuation of OA progression by miR-99a overexpression. (A) Histopathological staining of cartilage tissue from different groups of rats. (B) OARSI scores were determined by observation of Safranin O Fast Green staining sections. (C) The levels of pro-inflammatory cytokines (IL-1β, IL-6, and TNF-α) in blood samples derived from OA rats were determined by ELISA. (D) The levels of miR-99a and FZD8 expression in articular cartilage derived from OA rats. (E) The levels of Bax, Bcl-2, caspase-3, Col2A1, MMP-13, β-catenin, and FZD8 proteins in articular cartilage derived from OA rats were measured by western blotting. **p < 0.01, ***p < 0.001, compared with sham; ##*p* < 0.01, ###*p* < 0.001, compared with model, *N* = 10. AB, Alcian Blue staining; SF, Safranin O Fast Green staining; TBO, Toluidine Blue O staining

Our previous experiments demonstrated the suppressive effects of miR-99a on the in vitro model of OA. Subsequently, the miR-99a agomir was injected into the papain and L-cysteine-induced spines of OA rats. Four weeks after induction, the mean cartilage thickness was significantly decreased and the mean OARSIs score was increased by the papain mixture solution, while those effects were attenuated by injection of the miR-99a agomir (Fig. 5A-B). ELISA assays were conducted to evaluate the inflammation status. As shown in Fig. 5C, miR-99a agomir-treated OA rats had lower levels of IL-1β, IL-6, and TNF-α when compared with the model groups. Next, the relative levels of miR-99a and FZD8 expression in the articular cartilage of OA rats were analyzed. After confirming an inhibition of miR-99a expression in the model group and an overexpression of miR-99a in the model + miR-99a group, we observed a downregulation of FZD8 mRNA induced by miR-99a overexpression in the articular cartilage of OA rats (Fig. 5D). Consistent with the in vitro data, western blot results showed that miR-99a overexpression reduced the levels of Bax, caspase-3, MMP-13, β-catenin, and FZD8 proteins, but increased the levels of Col2A1 and Bcl-2 proteins in the model group (Fig. 5E).

## Discussion

As one of many gene regulators, microRNAs play an important role in osteoarthritis by several mechanisms, such as regulating bone metabolism, inflammation, chondrocyte injury, and extracellular matrix degeneration [29]. In our study, miR-99a was found to be expressed at low levels in severe spine OA, suggesting that miR-99a might be correlated with the degree of injury in OA. It is generally accepted that OA is caused at least in part by degeneration of articular cartilage resulting from an inflammation-induced upregulation of catabolism in chondrocytes [30]. An in vitro inflammation model created by stimulation with IL-6/TNF-α was used to explore the function of miR-99a, from which miR-99a was found to regulate chondrocyte viability, apoptosis, and extracellular matrix degeneration. In addition, an experimentally induced rat OA model also confirmed the above results.

The role played by miR-99a in regulating inflammation and cell apoptosis has been previously studied. Literature reports shows that miR‐99a is important for adipose tissue inflammation [14] and endothelial inflammation in cardiovascular diseases [31]. Furthermore, miR-99a was reported to regulate apoptosis in a variety of malignant cancers, including urinary bladder urothelial carcinoma [32], osteosarcoma [33], and non-small cell lung cancer [17]. RNA sequencing studies revealed that miR-99a was downregulated in the synovial fluid from an animal model of early OA [34], while its function and mechanism have never been explored. In our study, overexpression of miR-99a significantly suppressed chondrocyte apoptosis and cartilage ECM degradation in vitro and in vivo, and also reduced cartilage injury and the release of inflammatory factors induced in an OA rat model in vivo. Based on these findings, we speculate that miR-99a might serve as a biomarker for OA.

It is well known that miRNAs regulate biological functions primarily by inhibiting the expression of their target genes [35]. Here, we further confirmed that *FZD8* was a direct target of miR-99a. Furthermore, we found that overexpression of FZD8 significantly abrogated the suppressive effects of miR-99a on IL-6 and TNF-α‐induced extracellular matrix degradation and apoptosis, and also activated β-catenin. β-catenin is a key molecule in the canonical Wnt signaling pathway. A significant upregulation of β-catenin in articular cartilage causes cartilage degeneration involved in OA progression [24]. Inhibition of the Wnt/β-catenin pathway has been demonstrated to ameliorate the development of OA in a murine model [36]. FZD8, a G protein-coupled receptor protein belonging to the Frizzled family, has been recently reported to exert a biological function via the β-catenin pathway [37]. Collectively, our results suggest that overexpression of miR-99a might alleviate cartilage injury in experimentally induced spine osteoarthritis via the FZD8/β-catenin pathway.

Although miR-99a has been confirmed as a regulator of OA, its use in the clinic lies in the distant future. This is because microRNAs regulate a wide variety of genes, and their clinical use may produce undesirable side effects. Additionally, the possible occurrence of adverse immune reactions and the development of an appropriate drug delivery system must be carefully considered. Thus miR-99a needs to be more thoroughly investigated prior to its clinical use.

## Conclusions

In conclusion, our results showed that miR-99a reduced chondrocyte apoptosis and extracellular matrix degradation both in vivo and in vitro by targeting FZD8, suggesting miR-99a as a novel molecular target for treating spine OA.

## Supplementary Information

**Additional file 1:** 

## Data Availability

All data generated or analyzed in this study are available in the published article.

## Abbreviations

OA: Osteoarthritis
FZD8: Frizzled 8
IL-1β: Interleukin-1 beta
Runx2: Runt-related transcription factor-2
BMPR2: Bone morphogenetic protein receptor type 2
TNF: Tumor necrosis factor
MSC: mesenchymal stem/stromal cells
