# Supplementary material for: MiR-99a alleviates apoptosis and extracellular matrix degradation in experimentally induced spine osteoarthritis by targeting FZD8
Source: BMC Musculoskelet Disord. 2022 Sep 20;23:872. doi: 10.1186/s12891-022-05822-8 (PMC9487131; doi:10.1186/s12891-022-05822-8)
Supplement: Supplementary file 1 — Additional file 1: [file 12891_2022_5822_MOESM1_ESM.pdf]

Figure 2 Blots

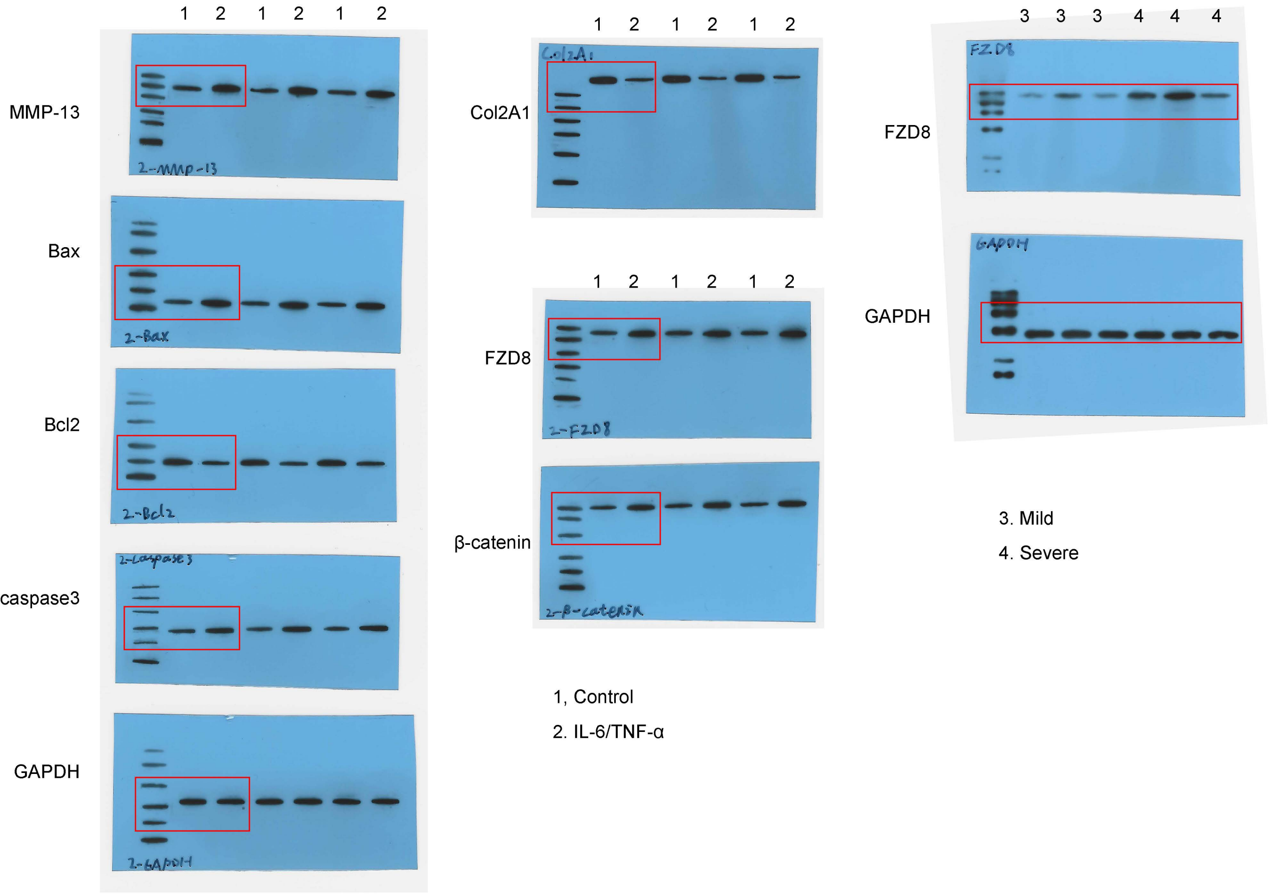

Figure 3 Blots

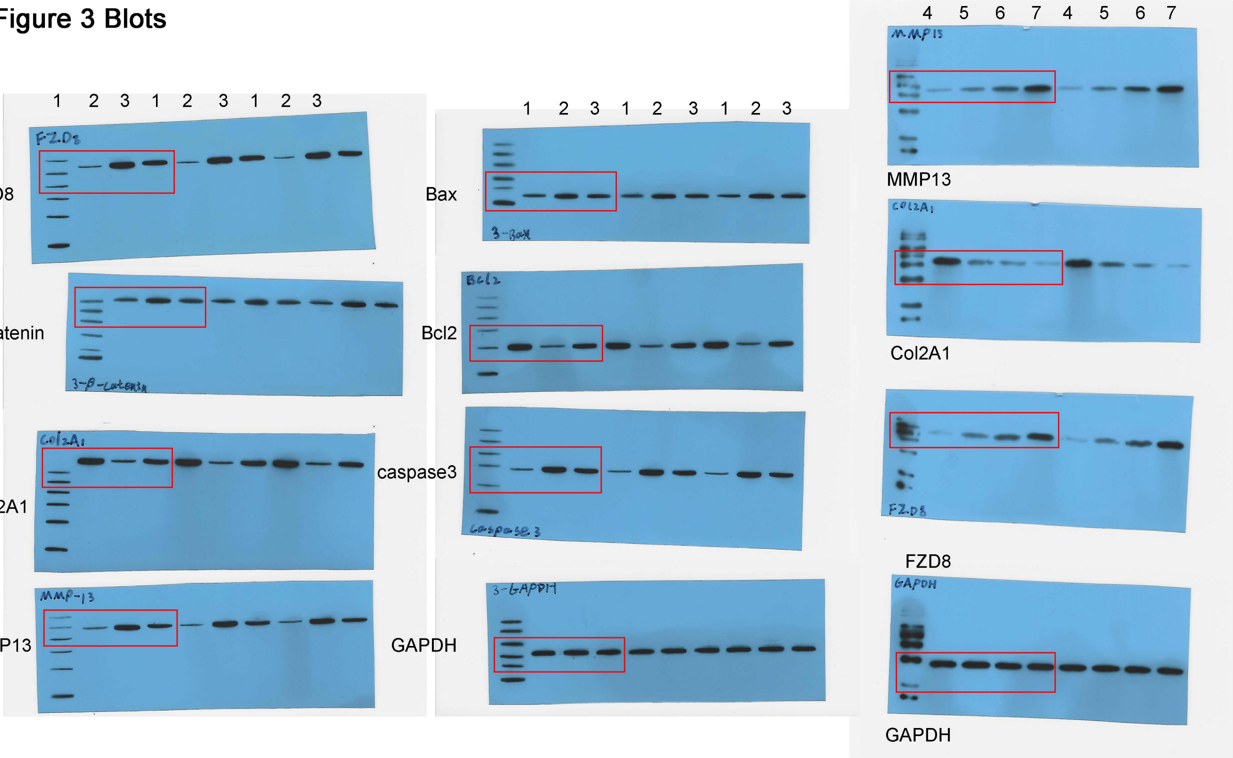

1. Control+Scramble
2. IL-6/TNF-α+Scramble
3. IL-6/TNF-α+miR-99a

4. Blank+NC
5. Blnak+antagomir
6. IL-6/TNF-α+NC
7. IL-6/TNF-α+antagomir

Figure 4 Blots

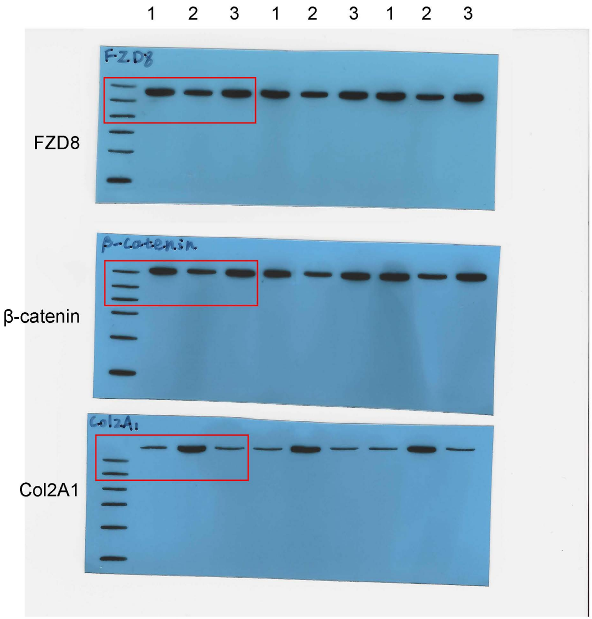

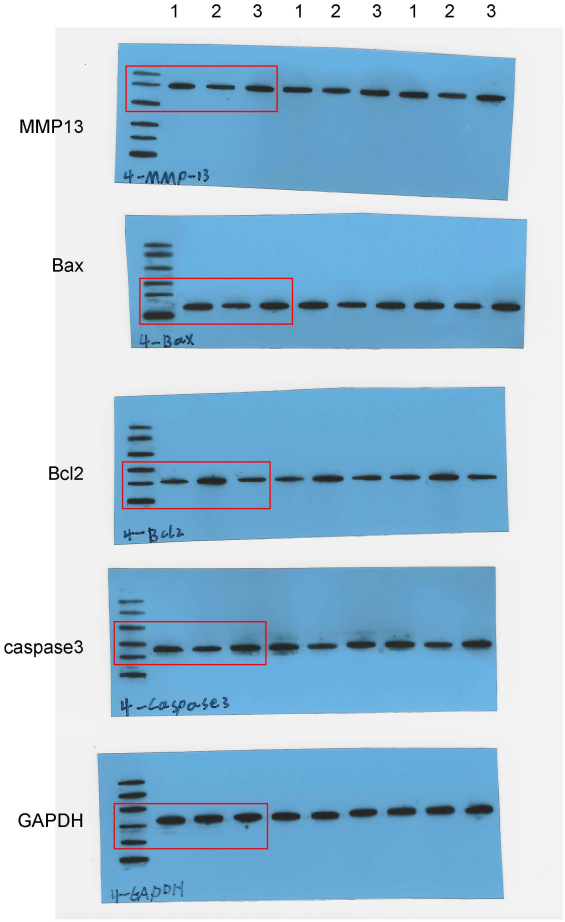

1. Vector
2. miR-99a
3. miR-99a+FZD8

Figure 5 Blots

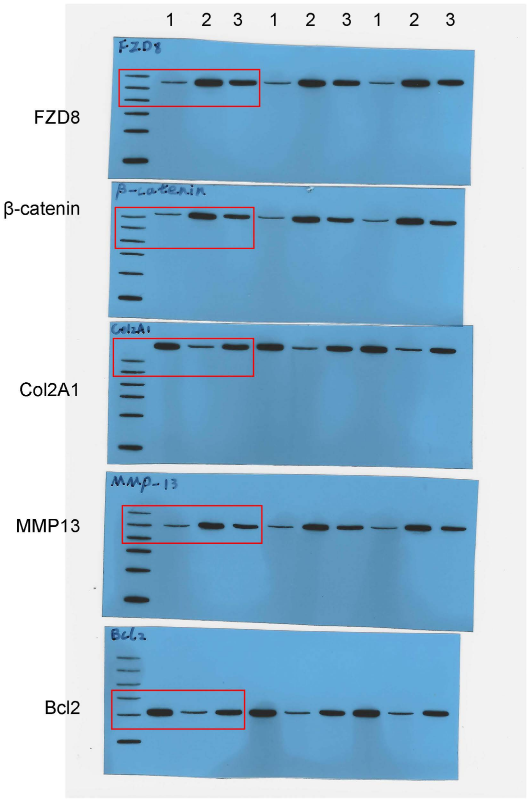

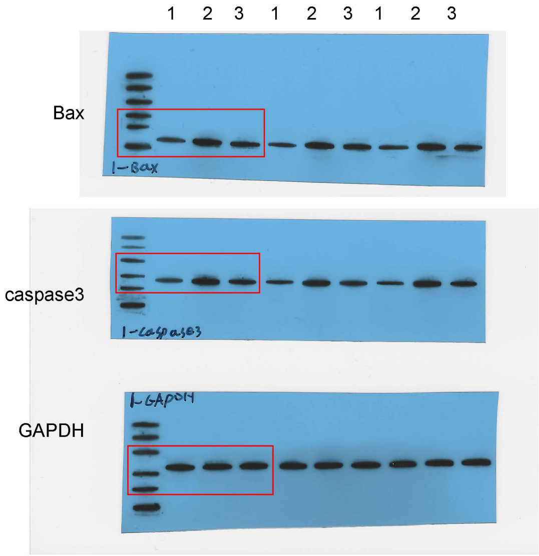

1. Sham
2. Model
3. Model+miR-99a
